# ℮-conome: an automated tissue counting platform of cone photoreceptors for rodent models of retinitis pigmentosa

**DOI:** 10.1186/1471-2415-11-38

**Published:** 2011-12-20

**Authors:** Emmanuelle Clérin, Nicolas Wicker, Saddek Mohand-Saïd, Olivier Poch, José-Alain Sahel, Thierry Léveillard

**Affiliations:** 1 INSERM, U968, Paris, F-75012, France; 2 UPMC Univ Paris 06, UMR-S 968, Institut de la Vision, Paris, F-75012, France; 3 CNRS, UMR- 7210, Paris, F-75012, France; 4 IGBMC, Laboratoire de Bioinformatique et Génomique Intégratives, 67404 Illkirch, France

## Abstract

**Background:**

Retinitis pigmentosa is characterized by the sequential loss of rod and cone photoreceptors. The preservation of cones would prevent blindness due to their essential role in human vision. Rod-derived Cone Viability Factor is a thioredoxin-like protein that is secreted by rods and is involved in cone survival. To validate the activity of Rod-derived Cone Viability Factors (RdCVFs) as therapeutic agents for treating retinitis Pigmentosa, we have developed e-conome, an automated cell counting platform for retinal flat mounts of rodent models of cone degeneration. This automated quantification method allows for faster data analysis thereby accelerating translational research.

**Methods:**

An inverted fluorescent microscope, motorized and coupled to a CCD camera records images of cones labeled with fluorescent peanut agglutinin lectin on flat-mounted retinas. In an average of 300 fields per retina, nine Z-planes at magnification X40 are acquired after two-stage autofocus individually for each field. The projection of the stack of 9 images is subject to a threshold, filtered to exclude aberrant images based on preset variables. The cones are identified by treating the resulting image using 13 variables empirically determined. The cone density is calculated over the 300 fields.

**Results:**

The method was validated by comparison to the conventional stereological counting. The decrease in cone density in *rd1 *mouse was found to be equivalent to the decrease determined by stereological counting. We also studied the spatiotemporal pattern of the degeneration of cones in the *rd1 *mouse and show that while the reduction in cone density starts in the central part of the retina, cone degeneration progresses at the same speed over the whole retinal surface. We finally show that for mice with an inactivation of the Nucleoredoxin-like genes *Nxnl1 *or *Nxnl2 *encoding RdCVFs, the loss of cones is more pronounced in the ventral retina.

**Conclusion:**

The automated platform ℮-conome used here for retinal disease is a tool that can broadly accelerate translational research for neurodegenerative diseases.

## Background

Retinitis pigmentosa (RP) is characterized clinically by an initial loss of night vision resulting from the degeneration of rod photoreceptors directly due to a genetic deficit, followed irreparably over a period of several years by the loss of central vision that results from the non-cell autonomous death of cone photoreceptors [1]. Because cones dominate the centre of the retina and are responsible for the high-acuity and color vision, their preservation would be medically relevant as a therapy aimed at preventing blindness [2]. We have studied the mechanisms involved in the secondary degeneration of cone photoreceptors in the *rd1 *mouse model of recessive RP, which carries a mutation in the rod photoreceptor-specific cGMP phosphodiesterase β-subunit gene [3]. Initially, we showed that grafting normal photoreceptors (97% of rods) into the eyes of this rod-less model, before the degeneration of cones, exerts a protective effect on cones [4]. We subsequently demonstrated that the neuroprotective activity was mediated by protein(s) secreted by rods [5,6]. One of these proteins, Rod-derived Cone Viability factors (RdCVF) whose expression is rod-dependent, was then identified by screening a retinal cDNA library in an assay based on the viability of cone-enriched cell culture cells made from chicken embryos [4]. The viability of these cells was monitored by fluorogenic probes for more than 200,000 cultures in a 96 wells format using a platform developed for this high content screening. RdCVF protein, when injected into the subretinal space of a rodent model of retinal degeneration prevents the loss of function of cone photoreceptors [4,7]. Interestingly, RdCVF is encoded by the Nucleoredoxin-like gene, *Nxnl1 *that belongs to the family of thioredoxin proteins, reducing oxidative stress, a condition encountered broadly in neurodegenerative diseases [8]. This novel trophic signaling is part of an endogenous defense response since cone photoreceptors degenerate during aging in the *Nxnl1-/- *mouse and at an accelerated rate in the presence of high levels of oxygen [9]. We have also identified *Nxnl2*, a paralogue gene that also encodes a cone survival factor, RdCVF2 [10]. The administration of RdCVF in patients suffering from RP at early stage of the disease could therefore reduce secondary cone degeneration and prolong central vision.

Whatever the delivery system used--be it protein injection, viral vector delivery or even by reactivation of the RdCVF promoter in neighboring cells [7,11,12] --this translational research program requires the development of a robust system to test the trophic activity toward cones of the therapeutic molecules produced for human clinical trial. The technical difficulties in measuring accurately the cone density as noticed by LaVail et al., [13] were solved using stereological counting [5]. We report here the development of ℮-conome, a fully automated platform for measuring cone density in mouse models of cone degeneration. We demonstrated the reliability of this platform by comparing the kinetics of cone degeneration in the *rd1 *retina measured using our platform to that measured by standard stereological counting. In parallel, we developed an automated, operator-independent stereological method that was also evaluated for accuracy. The automated platform scanned the whole surface of the flat-mounted retina allowing the user to evaluate the local density of cones as demonstrated by the pattern of S-cones in the *rd7 *retina. We studied the spatiotemporal pattern of cone loss in the *rd1 *retina as well as the loss of cones in the nucleoredoxin-like gene knock-out mice *Nxnl1-/- *and *Nxnl2-/-*, revealing a more pronounced loss in the ventral region of the retina. The development of the platform ℮-conome, as validated in the experiments reported here, will accelerate the translational research required to evaluate RdCVF as a therapeutic agent for cone degeneration in RP.

## Methods

### Animals

All procedures were in compliance with the Association for Research in Vision and Ophthalmology Statement for the use of Animals. The protocols approved by the National Eye Institute Animal Care and Use Committee. Animals, mixed gender were housed under a 12 hours light/12 hours dark cycle and given *ad-libitum *access to food and water. The two congenic C3H lines [14] (C3H*rd1/rd1 *and C3Hwt/wt) were re-derived and maintained at Charles River. The *rd7 *colony was obtained from Jackson Laboratories, (USA). The homozygous knockout mice *Nxnl1 *and their controls are described in [9]. The *Nxnl2 *line is described in Jaillard *et al*. (manuscript in preparation). All three lines have a pure BALB/c background. Day of birth was designated postnatal day 0 (PN0).

### Tissue collection

Retinal tissues were obtained from C3Hwt/wt and C3H*rd1*/*rd1 *eyes aged PN15 to 90 days. All the right eyes of each genotype (n = 7) per age (PN15, 35, 43, 60 and 90 days) were analyzed by automated counting and the left eyes by stereological counting. We then replicated the experiment by inverting eye polarity. Animals were sacrificed by decapitation. The orientation of the eyes was marked on the limbus at 12 O'Clock position before enucleating. Neural retinas were dissected in phosphate-buffered saline (PBS) at room temperature from the posterior eyecup followed by immersion in cold 4% paraformaldehyde (PFA) in PBS at pH 7.4. For cryosectioning, eyes were fixed in cold PFA 4% in PBS overnight after puncturing the cornea then left 1 hour in 10%, 11/2 hour in 20% and 3 hours in 30% sucrose then quickly embedded in 4% gelatin with liquid nitrogen. The section plane was extended along the vertical axis from the optic nerve head in the posterior retina to the cornea.

### Production of polyclonal antibodies against S-Opsin

A peptide corresponding to the second extracellular loop of mouse short wave opsin (CGPDWYTVGTKYRSE) was synthesized and coupled to ovalbumin and injected into two New-Zealand rabbits. The specificity of the polyclonal antibodies was confirmed by immunohistochemistry and compared with the pre-immune serum on *rd7 *retina (not shown).

### Immunohistochemistry on flat-mounted retina

Fixed retinas were rinsed with PBS three times, before permeabilization (30 seconds for all genotypes and 5 minutes for C3H*rd1/rd1*) in PBS containing 0.1% Triton X-100, followed by incubation 1 hour in blocking buffer PBS containing 1% of bovine serum albumin (BSA), 0.1% Tween-20 and 10% normal goat serum (NGS). After washing, retinas were labeled for 3 hours at room temperature or overnight at 4°C with Alexa Fluor 594-coupled peanut agglutinin lectin from arachis hypogae (PNA) [15] (1:40, Invitrogen, USA). For S-Opsin, the retinas were incubated with anti-S-Opsin (1:400) 4 hours at 37°C followed by incubation overnight at 4°C in the same blocking buffer. The retinas were washed six times with blocking buffer without NGS and incubated with secondary antibody, goat anti-rabbit IgG conjugated to Alexa Fluor 488 (1:400, Invitrogen, USA). We then performed four incisions of the whole retina to obtain flat-mounted retina with the photoreceptor layer facing up. Four retinas were placed on a vertical line. For automated acquisition, the first and fourth retinas were kept at a distance of 5 mm from the upper and lower edges of the slide respectively.

### Immunohistochemistry on frozen eye sections

Ten μm thick frozen eye sections were dried at room temperature for immunohistochemistry as previously described [9] with few modifications. No permeability was applied and PNA was added for 3 hours at room temperature in the blocking buffer before immerging slides into blocking buffer with Dulbecco's Modified Eagle Medium (DMEM), 4.5 g/l of glucose, 10% of NGS and 10% of fetal calf serum, 1 hour at room temperature followed by S-Opsin incubation in the same buffer during 3 hours at room temperature. After washing, the nuclear marker 4'-6-diamidino-2-phenylindole (1:1000, DAPI, Sigma) was added with the secondary antibody. Finally the sections were mounted on glass slides with fade-resistant mounting media (Biomeda, Forster city, CA) topped with coverslip and imaged with a fluorescent microscope (Leica, USA).

### Automated image acquisition of labeled cones on flat-mounted retina

**Figure 1:**
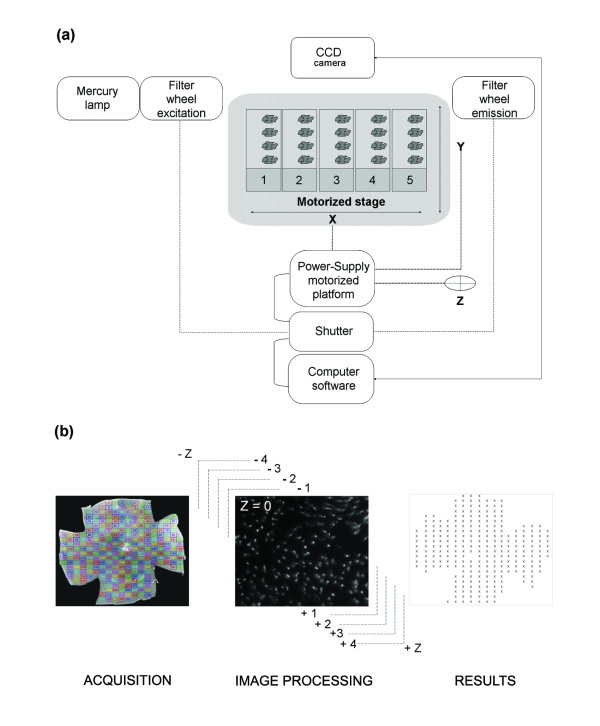
**℮-CONOME, the automated microscopy platform**. **(a) **Connections between the different pieces of equipment. The motorized stage is made for five slides with a maximum of four retinas per slide. **(b) **Visualization of the grid used for acquisition and counting (left). Representative image of an acquisition for one field and the nine Z-planes (centre), the illustration corresponds to the best focus Z = 0 as indicated. Schematic representation of the results of global automated counting in an Excel sheet (right). The color code is providing an easy visualization of the fields of the grid during acquisition.

An inverse microscope (Nikon, Eclipse TE200, and 2000) was equipped with a mercury super high pressure lamp, a computer driven motorized stage (Multicontrol 2000, Martzauzer, Wetzlar), an optical filter switch (Lambda 10-2, Shutter Instrument company) for two excitation filters (485 and 520 nm) and two emission filters (520 and 635 nm), a shutter driver (JML Direct Optics), two objectives 4X (0.10, infini/- , WD 30) and 40X (Plan Fluor 40X/0.75 Dic M, infini/0.17, WD 0.72), and a CCD camera (CoolSNAP FX and HQ, Photometrics). The pieces of equipment were connected (Figure 1) and controlled by algorithm (Additional file 1) developed with Metamorph (Universal Imaging Corporation, Sunnyvale, CA, USA). A maximum of 5 slides with 4 flat-mounted retinas were placed on the motorized stage platform and the position of the centre of the retina were set-up manually using a 4X objective to record the coordinates (X, Y). The objective was switched to 40X and the platform stage moves back to the first retina and the focus manually set-up on labeled cones and recorded (Z). The program offers two options for acquisition: the entire retina (grid) or a draughtboard grid. The acquisition grid was designed from nine assembled images (4X objective) and used for the acquisition (X40 objective) of ~300 fields (Figure 1) of identical surface area (0.0376 mm^2^), covering the entire surface of the retina (CoolSNAP FX: 217.75 × 172.53 μm, CoolSNAP HQ: 224.46 × 167.70 μm). Two incremented stages of autofocus were executed to recover the focal plane for each individual plane (~300) on the overall retinal surface. The first stage scans six different planes within the depth of the retina with an increment of 60 μm approaching the focal plane. The second stage is a fine adjustment from this position using 8 planes with a lower increment of 15 μm. Centered on this focal position, a stack of nine images per field distanced by 0.7 μm were acquired in the Z-direction (Figure 1). All the stacks were registered in Tagged Image File Format (TIFF) in a file named by the date of the day followed by an increment numbers. All images had a resolution of 650 × 515 pixels in 16-bits images (1.72 Gigabytes for the entire retina).

### Global automated cone quantification

The stacks of images were treated with an algorithm using Metamorph to quantify cones (Additional file 1). A projection though the nine plans of each file was applied in order to reveal best focus plane to which was applied an auto-threshold to reduce the background. The algorithm is not perturbed by the overall orientation and twist of the cones due to the use of a morphological filter. In addition, the projection through the 9 planes that is operated before counting is creating a virtual image in which the cones are preferentially seen as a transversal section though the cell body. This threshold was used to detect dark areas resulting from retina pigmented epithelium residue and to exclude the images that do not fulfill the preset criteria. When this phenomenon is predominant, the field is excluded using the function dark_max (Additional file 1b, line 11 in Editor\variables). The image resulting from one field is segmented and the threshold is applied locally to overcome difference in brightness within an image (Additional file 1b, line 12-14 in FINDSPOT\3D Measurements). An adjustment of digital contrast was operated to discriminate cells among the cluster from background. At this stage eleven ordered variables empirically determined were called [best focus average intensity, autothreshold area %, number of cells detected on best focus before treatment, minimal intensity, number of minimal cells, max background, cluster, dark max, spot cut-off, spot size, spot surface, Additional file 1] to filter the images to be counted. The variables are: lane 1) Best Focus average intensity, average intensity of the projection of the nine images; 2) Percentage Best Focus. Threshold autothreshold area percentage, percentage of cells within the projected image over the threshold; 3) Number of cells detected with Best Focus before treatment, before the treatment with the algorithm findpot 4) Spotcutoff, the cutoff that is used to exclude objects with aberrant measures; 5) Spotsize, the size above which objects are excluded; 6) SurfSpot, the surface above which objects are excluded; 7) IntMin, the minimal intensity for an object to be retained; 8) NBObjectsMin, the minimal number of objects that should contain a projected image; 9) FondMax, the maximal intensity of the total space between the objects; 10) Cluster, The size of an object that is rejected since it is considered as a cluster of cells; 11) Dark_max, the minimal intensity of a projected image. The values above the threshold were arbitrarily equaled to zero in order to enhance the contrast. The function find-spot was used on each image filtered with the eight fits variables, to dissociate neighboring cells and by calling three variables: the spot cut-off, the spot size and the spot surface resulting in an accurate counting (Additional file 2). Finally, the number of counted cells in the selected image was mentioned with all parameters described above before closing it. The density of cones are locally associated with their coordinates (Figure 1), and used to calculate the density of cones over the surface of the retina after having removed the excluded fields.

### Stereological cone Quantification

The stereological counting was achieved as described previously [5,7]. Briefly, the cones were counted on 50-80, non-overlapping, 1,225 μm^2 ^fields selected with a systematic random sampling procedure applied to the retinal surface from the centre of the optic nerve head over a radius of 2 mm.

### Stereological automated cone quantification

The process was adapted from [5]. The area comprising the optic nerve is indicated by the operator for its exclusion. The number of fields was reduced from the global method by using a draughtboard grid. Within each field, a frame (30 μm × 30 μm) was drawn with exclusion of object in contact with one of both axes X and Y. Within these fields, the cones were counted with the parameters used for the global methods. The results are expressed as the average cone density over the counted fields.

### Spatial distribution

The spatial distribution of cones was inferred from the local densities with their coordinates X, Y recorded in an Excel file and the orientation (Ventro-Dorsal, Naso-Temporal). A virtual eye fundus representation was designed to visualize the data. The value X and Y were used to calculate the position of the optic nerve and the local density of cones in 9 rows taken from this centre are projected on the disk annulus by annulus. To represent the amount of cones in each segment, a color scale was applied using multiples of 45 (45, 90,......,405).

### Statistical Analysis

Statistical analyses were performed with GraphPad Prism5.0, with *p *< 0.05 considered significant. Unpaired Student's t-test was used to compare density of cones levels in *rd1 *at different ages with a Welch's correction. In figure 4, we compared the value between three groups, by a two-way anova where one factor stands for the days and the second for the technique. For the normalized test using the quantile-quantile normalization [16], the quantiles of the set of measures obtained by each technique are identical. To apply this normalization while avoiding any bias due to overrepresentation of some dataset, curves have been made first comparable by resizing each set in such a manner that for a dataset the number of measurements is equal using each method. As this can be done in several ways by taking out 4 values, (one for PN60 for global automated method, one for PN60 and for PN90 for stereological method and one for PN35 for automated stereological method), all 2,353 possible combinations were tested. For each combination the two-way Anova led to a p-value of one after normalization indicating that the there is no difference between the three methods. To test for cone regionalization in figures 7C and 7E, a paired t-test was used to compare the means cone density (PNA) from the same retina of the dorsal and ventral region for *Nxnl+/+ *(n = 10), *Nxnl1-/- *(n = 7) and *Nxnl2-/- *(n = 8) mouse. To compare the means S-cone density (S-opsin) from the same retina of the dorsal and ventral region for *Nxnl+/+ *(n = 5) and *Nxnl2-/- *(n = 5) mouse.

## Results

### Development and validation of the automated counting platform

**Figure 2:**
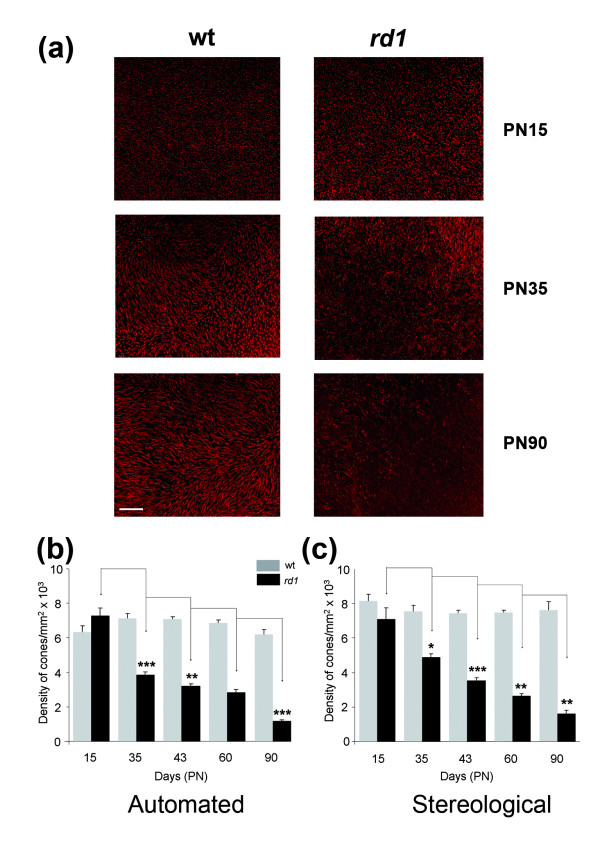
**Validation of the automated counting method**. **(a) **Staining of cones with PNA in wild-type (wt) and in retinal degeneration (*rd1*) of flat-mounted retina at PN15, 35 and 90. **(b) **Average cone densities of *rd1 *and wt retinas using global automated counting method. **(c) **Average cone densities of *rd1 *and wt retinas using stereological counting on contralateral eyes. The data represents an average of at least seven retinas per age and per genotype. Error bars show +/- SEM, * p < 0.05, ** p < 0.01, *** p < 0.001. Scale bar: 100 μm.

An inverted fluorescent microscope, motorized and coupled to a CCD camera was designed to automatically record images and count cones labeled with fluorescent peanut agglutinin lectin (PNA) [15] on flat-mounted retinas (Figure 1). In an average of 300 fields per retina, nine Z-planes at magnification X40 and separated from each other by 0.7 μm were acquired after two-stage autofocus individually for each field, and covering the entire thickness of outer retina. The stack of 9 images (650 × 515 pixels) was used to create a projection that was subject to a threshold, filtered to exclude aberrant images (based on preset variables) and contrast adjusted. The cones were identified by treating the resulting image using 13 variables empirically determined (see Methods). The cone density was calculated over the ~300 fields, and averaged over the counted fields. This method was compared to a non-automated approach used in previous studies [5]. We validated the platform by studying the kinetics of secondary cone degeneration in the *rd1 *mouse model [17]. For both the *rd1 *(C3H*rd1/rd1*) and wild-type (C3Hwt/wt) maintained on identical genetic background [14], the right eyes (n = 7) at post-natal days (PN) 15, 35, 43, 60 and 90, were analyzed with the novel automated counting method and the left eyes used for stereological counting [5]. The eyes were marked at the 12 O'Clock position before removing the neural retina in order to preserve the orientation. The decrease in cone density from PN15 to 90 was thus calculated from the PNA-labeled (both S- and M-cones) flat-mounted *rd1 *retinas (Figure 2a). PNA labels the extracellular sheaths around their outer segments and consequently is an indirect measurement of cones. It is nevertheless extensively used. As expected, no differences in cone density were found for the wild-type (wt) mouse using either methods (Figure 2b, 2c and Additional file 3). At PN15 the values for *wt *and *rd1 *are not statistically different even if the remaining rods at this age might interfere with the automated counting of cones. When considering the loss of cone in the *rd1 *retina, the results obtained were quite similar for both methods. The only noticeable difference was observed at PN35 with a reduction of the cone density of 49% (Figure 2b) versus 31% (Figure 2c), which does not result from a bias in the sampling since the equivalent results were obtained after having reversed the polarity of the eyes (Additional file 3).

**Figure 3:**
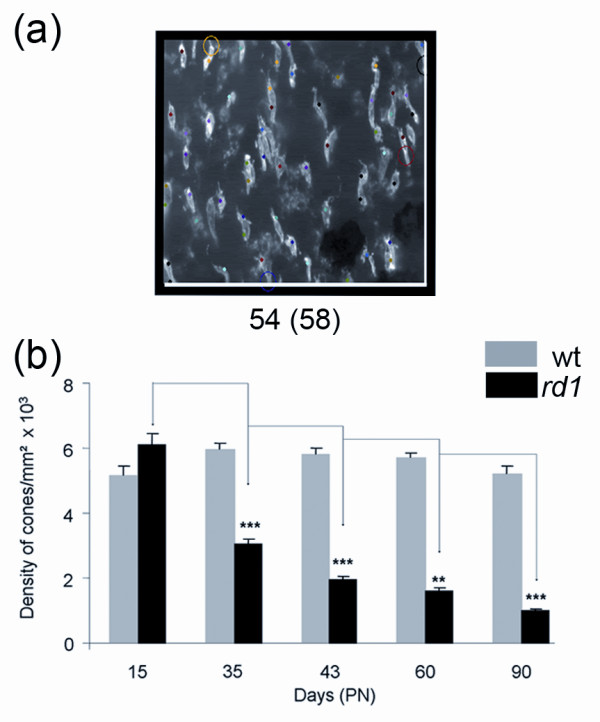
**Automated stereological counting method**. **(a) **A counted area showing the cones. The number of cones after exclusion and before exclusion (in brackets) is mentioned below the image. Cones in contact with the two coordinates (white lines) are automatically excluded. The colored dots represent the increment of the counting process. The colored circle point to excluded objects following the counting parameters (Spotcutoff, Spotsize and SurfSpot, Additional file 4) and counting window. More specifically, from the top to the bottom, the circles mean: Yellow, not satisfying the counting parameters; Black, cross the Y exclusion axe; Red, not satisfying the parameters and Purple, not satisfying the counting parameters. **(b) **Average cone densities of *rd1 *and wt retinas using automated stereological counting (n = 7). Error bars show +/- SEM, * p < 0.05, ** p < 0.01, *** p < 0.001.

**Figure 4:**
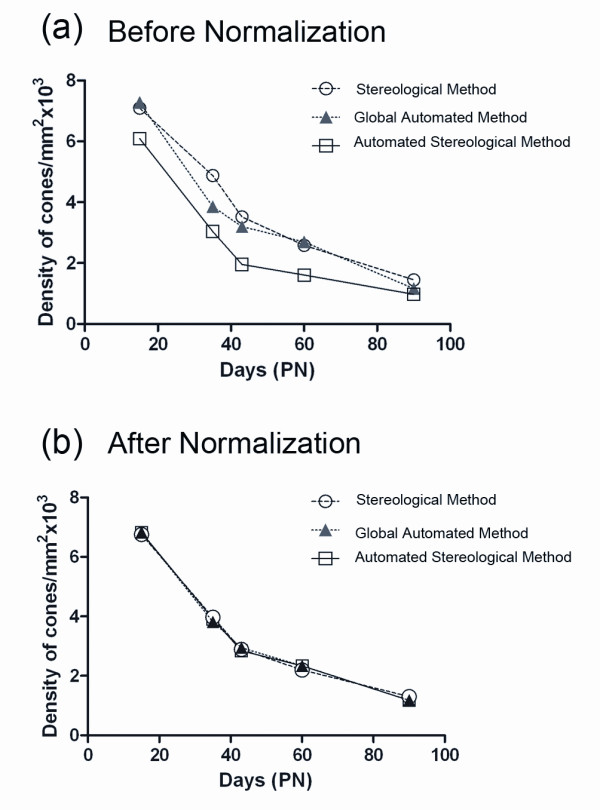
**Comparison of the three methods of counting**. **(a) **Average cone density before normalization for the three methods used (stereological, global automated and automated stereological method). A two-way ANOVA with one factor representing days and the second factor the method, shows a difference with a p-value equal to 6.42 10^9^. **(b) **Average cone density after normalization for the three methods used. The normalization involved use of the quantile-quantile method so that the quantiles of the set of measures obtained by each technique are equal. This normalization revealed a p-value of 1 indicating the absence of difference between the three methods.

We also developed an automated stereological counting method based on smaller fields (218 × 173 pixels) distributed over the surface of the retina. We expected that the automated identification of cones from these reduced fields would be facilitating the recognition of cones by the software (Figure 3a). When this method was applied to the images used in figure 2b, similar results were observed with a reduction of 50% of the cone density between PN15 and 35 for the *rd1 *retinas, while the overall densities were slightly reduced (Figure 3b, Additional file 3). The loss of cones between PN15 and 35 in the *rd1 *retinas seems to be distinct for both automated methods when compared to stereology (Figure 4a). However, when the data were normalized using the quantile-quantile method [16] the counts look very similar, yielding a two-way ANOVA value of 1 indicating that the difference most likely results from a systematic offset (Figure 4b and Additional file 4). This indicates that the two automated methods measure the cone density with equivalent accuracy as the more tedious stereological one.

### Spatiotemporal loss of cones degeneration in the *rd1 *mouse

**Figure 5:**
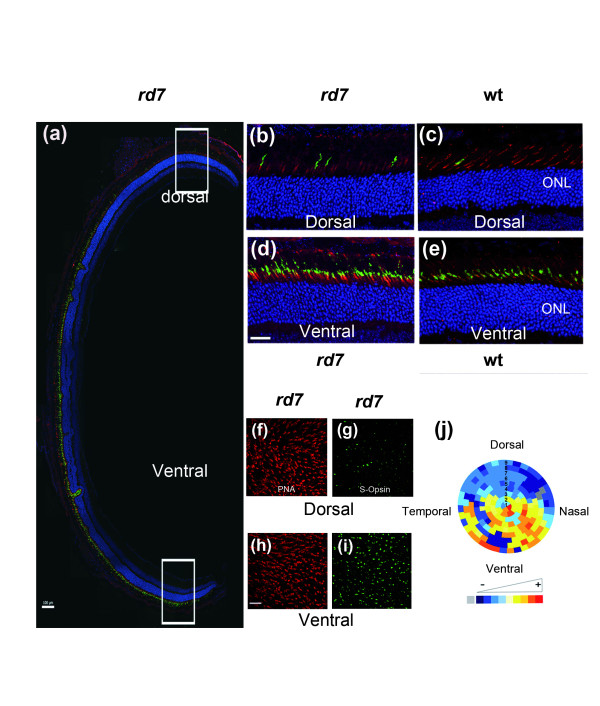
**Regionalization of S-cones in *rd7 *retina**. **(a) **Staining of PNA (red), S-Opsin (green) and nuclei (blue) on a section of *rd7 *retina at PN35 (Scale bar: 100 μm). **(d, b) **Higher expression of S-opsin ventrally than dorsally at higher magnification. **(e, c) **The difference of expression of S-Opsin expression in wild type (C3HfHeA) at high magnification. Staining of PNA **(f, h) **and S-Opsin **(g, i)** in *rd7 *flat-mounted retina at PN35. Scale bar: 25 μm for b-i. **(j) **Virtual eye fundus representing the S-cone density in the *rd7 *retina in 9 rows from the centre (optic nerve). The density of S-cones in each segment is encoded by a color scale. Excluded areas are represented by a grey color and the absolute cone density value from dark blue, yellow to dark red are: 45, 90, 135, 180, 225, 270, 315, 360 and 405 cones/surface unit.

The global automated counting method on oriented flat-mounted retina provides the opportunity to study the spatial distribution of the cones. We explored this potential by measuring the S-cone densities in the *rd7 *retina. The *rd7 *mouse carries a recessive mutation in the orphan nuclear receptor *Nr2e3 *causing a developmental defect that results in an excess of S-cones [18]. The S-cones were labeled with anti S-opsin polyclonal antibodies (Figure 5a). The excess of S-cones are localized in the ventral part of the rd7 retina (Figure 5d and 5b), when compared to the wild-type on a C3HfHeA (Figure 5e and 5c), and on a C57BL/6@N background (Additional file 5). This excess of S-cones was also observed on flat-mounted *rd7 *retinas and similarly localized to the ventral part of the retinal explant (Figure 5i and 5g), however there is no apparent difference in the total number of cones, labeled with PNA (Figure 5h and 5f). After global automated counting, we reconstituted the density of S-cones on a virtual eye fundus of the *rd7 *mouse in order to facilitate the visualization of the results (see Methods). In this representation, S-cones are concentrated in the ventral region of the *rd7 *retina (Figure 5j).

**Figure 6:**
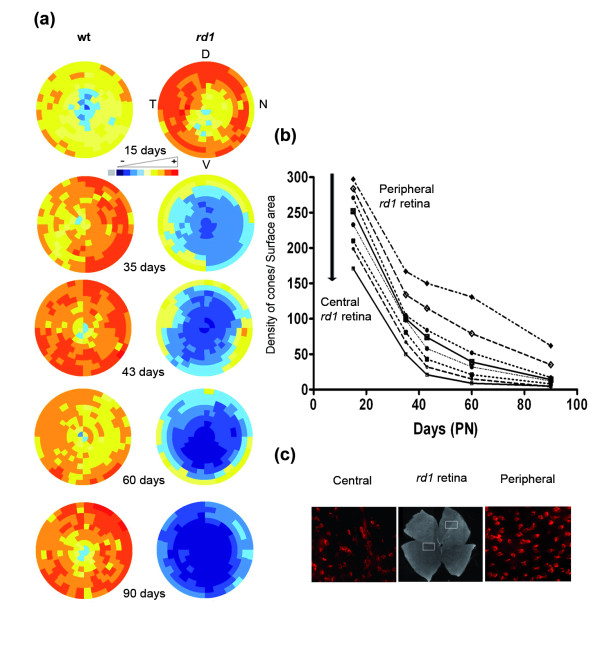
**Spatio-temporal quantification of cones of *rd1 *flat retina during cones degeneration**. **(a) **Virtual eye fundus representing the cone density measured by global automatic counting of cones labeled with PNA on flat-mounted wild-type and *rd1 *retinas from PN15 to PN90 (D: dorsal, V: ventral, T: temporal, N: nasal). The density of cones in each segment is encoded by a color scale and the absolute cone density value from dark blue, yellow to dark red are: 45, 90, 135, 180, 225, 270, 315, 360 and 405 cones/surface unit. **(b) **Average density of cones (cones/0.0376 mm^2^) in *rd1 *retina for the 9 rows centered on the optic nerve (peripheral to central) from PN15 to PN90. **(c) **PNA staining cones in *rd1 *flat-mounted retina at PN15 in central area of the retina (left) and peripheral area retina (right).

We next used this representation to visualize the spatiotemporal loss of cones in the *rd1 *retinas. The virtual eye fundus was obtained by averaging 7 retinas. For the wild-type mouse, an evenly distributed increase in cones was observed between PN15 and 35 accordingly to figure 2B, there-after no major change occurs (Figure 6a). The distribution of cones in the *rd1 *retina at PN15 is rather similar to the wt retinas at PN35. However, from PN15 to 90 a decrease of cones at the centre of the retina is observed. This is consistent with the centro-peripheral degeneration of cones as reported previously for *rd1 *[19-21], however it does not necessarily imply that the cones degenerate at a higher rate in the centre of the retina in a model of rod-cone interaction [22]. In order to address this question we plotted the average density of cones in the *rd1 *retina in concentric circles from the centre of the retina (Figure 6b). As seen on the graph, the loss of cone at each position relative to the centre is similar between PN15 to 45. Because the density of cones is higher in the periphery at PN15 (Figure 6c), the centro-peripheral pattern observed in figure 6a is created by the the centro-peripheral density of cones at start. This early phase correlates with the death of cones resulting presumably from the loss of expression of diffusible trophic molecules produced by rods [4,10]. We observed at latter stages, from PN35 that the cones degenerate at a slower pace. This means that at least two events are involved in the degeration of cones. The protective effect generate by antioxidant molecules [23] indicates that oxidative stress may be one of the triggers. RdCVF, the thioredoxin-like protein would be limiting oxidative damage that would be higher when rod die between PN15 and 35. From PN35 to 60 the two rows of cone at the periphery are retarded as compared to more central cones in agreement with earlier observations on persistence of cones in the periphery of *rd1 *retinas [19].

### Pattern of cone degeneration in the mouse lacking the nucleoredoxin-like genes

**Figure 7:**
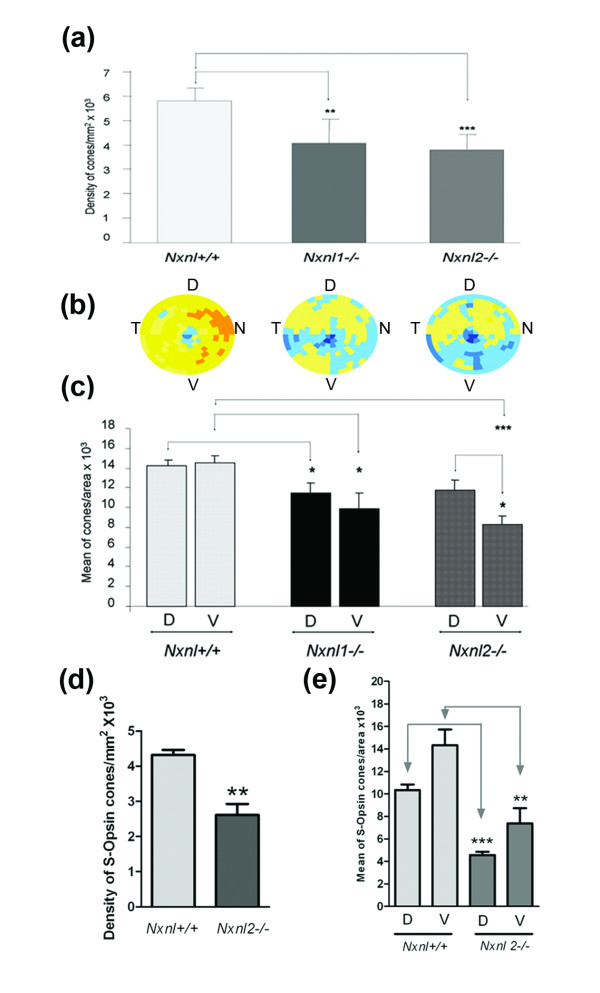
**Loss of cones in the *Nxnl1-/- *and *Nxnl2-/- *retinas its regionalization**. **(a) **Global automated counting of cones of *Nxnl1-/- and Nxnl2-/- *versus *Nxnl*+/+ at PN8 months, (n = 7, 8, 10 respectively). Error bars show +/- SEM, * p < 0.05, ** p < 0.01, *** p < 0.001. **(b) **Visualization using virtual eye fundus of the cone density for the three genotypes. The density of cones in each segment is encoded by a color scale. Excluded areas are represented by a grey color and the absolute cone density value from dark blue, yellow to dark red are: 45, 90, 135, 180, 225, 270, 315, 360 and 405 cones/surface unit. **(c) **Regionalization of the cone density in the ventral versus the dorsal region of the retina. The surface of the regions (2.48 mm^2^) represents 22% of the retina. Error bars show +/- SEM, * p < 0.05, ** p < 0.01, *** p < 0.001. **(d) **S-cone density on the *Nxnl+/+ *and *Nxnl2-/- *retinas. **(e) **Regionalization of the S-cone density in the ventral versus the dorsal region of the *Nxnl+/+ *and *Nxnl2-/- *retinas.

Since the secondary degeneration of cones results at least partly from the loss of expression of the Rod-derived Cone Viability Factors [8], we examined the spatiotemporal pattern of the cones in mice lacking either the nucleoredoxin-like 1 or 2, genes (*Nxnl1 *and *Nxnl2*, respectively). We have reported a reduction of 17% in cone number in the *Nxnl1-/- *mouse at 15 weeks of age [9]. Here we reported the progression of this degenerative process with a 30% reduction of cones at 8 months (Figure 7a). We also investigated the density of cones in the *Nxnl2-/- *mouse, the gene encoding the paralogue RdCVF2 [10] (Jaillard *et al.*, manuscript in preparation). At 8 months of age the cone density was found to be 34% reduced as compared to controls on identical genetic background. The distribution pattern of this decrease in cone density is not homogenous, with an apparent higher deficit in the ventral region of the *Nxnl2-/- *retina, compared to the *Nxnl1-/- *mouse (n = 7-10, Figure 7b). In order to quantify this regional effect we compared the density of cones in 66 fields (2.48 mm^2^) at ventral and dorsal locations (Figure 7c). The dorsal and ventral fields are equidistant from the optic nerve. The respective 66 fields represent 6 rows and 11 columns centered on the dorso-ventral axis. The dorsal fields are separated from the ventral fields by a two rows (335.4 μm). This analysis shows that there is a statistical difference in the deficit of the ventral versus the dorsal region of the retina for the *Nxnl2-/- *mouse, suggesting that the two paralogous genes are part of a slightly distinct endogenous neuroprotective signaling pathways. We have observed similar deficit in the S-cone density in the *Nxnl2*-/- retina compared to its control (n = 5, Figure 7d). The analysis of the regionalization shows that the S-cone are affected to the same extend in the dorsal and in the ventral region of the *Nxnl2*-/- retina (Figure 7e), demonstrating that both the M and the S-cones are affected by the disruption of the *Nxnl2 *gene.

## Discussion

We demonstrated the usefulness of ℮-conome, a novel automated platform to quantify cone survival in mouse models of retinal degeneration. This technology will greatly accelerate the transition form bench to clinic for therapeutic agents such as Rod derived Cone Viability Factors that are aimed at preventing vision loss in patients suffering from retinitis pigmentosa. The robustness of the platform relies on the segmentation of the flat-mounted retina into ~300 fields in which 9 z-planes are acquired from the best focus determined individually form each field. The treatment of each of these 9 images involved filtering steps which include a morphological filter and the identification of each object (cones) using parameters whose values have been empirically set-up. The trophic effects of RdCVF proteins formulated for clinical trial or of AAV-RdCVF [7,12] can now be quantified in a less time-consuming manner that avoids human bias, thus facilitating dose-response studies. This platform is essential for the transfer of this promising therapeutic approach into clinical practice. It also permits the measurement of local effects as demonstrated by the reduction of the cone density in the ventral part of the retina of the *Nxnl2-/- *mouse. This is important considering the desired diffusion of the trophic effect from the site of injection. It is interesting to notice that such gradient of cone degeneration was also observed in a mouse with a specific inactivation in cones of the regulatory subunit p85 alpha of PI3Kinase [24]. We also demonstrate that this method can be efficiently applied to other markers in addition to the lectin PNA. The polyclonal anti-S-opsin antibodies were used to quantify the excess of S-cones in the ventral part of the *rd7 *mouse model. The platform is also a tool to study the signaling between rods and cones [9]. It could also be used to quantify the up-regulation of HIF1A and GLUT1 in cones that is observed following rod degeneration [25].

The quantification of neuron survival and neuropathological lesions in neurodegenerating tissues is essential for the development of therapeutic strategies relying on the delivery of trophic factors [26]. With regard to other retinal diseases, ℮-conome was used to count retinal Brn3a-labeled cells ganglion cells by placing this cell layer in the direction of the objective and by recalculated the eleven parameters used (results not shown). In the brain, the automated stereological method developed to count cone photoreceptors could be applied to quantify ischemic brain injury using histological sections labeled to track apoptosis using terminal UTP nick end labeling (TUNEL). It should be noted that the high-throughput method developed for the quantification of neurofibrillary tangles and senile plaques found in the brains of patients affected by Alzheimer's disease involves a manual step of focusing the images which significantly slows the process [27], compared to the method used here.

## Conclusions

In summary, e-conome provided an accurate platform to measure the density of the cones. In addition we show that the spatiotemporal pattern of cone loss in the *Nxnl2-/- *retina proceeds from the ventral part. The automated platform used here for retinal disease could accelerate translational research for neurodegenerative diseases more broadly.

## List of abbreviations

BSA: Bovine serum albumin; DMEM: Dulbecco's Modified Eagle Medium; NGS: Normal goat serum; *Nxnl1: *Nucleoredoxin-like 1 gene; *Nxnl2*: Nucleoredoxin-like 2 gene; PBS: Phosphate-buffered saline; PFA: Paraformaldehyde; PN: Post-Natal day; PNA: Peanut agglutinin lectin from arachis hypogae; RdCVF: Rod-derived Cone Viability factor; TUNEL: Terminal UTP nick end labeling

## Competing interests

T.L. and J-A.S. are patent-holders on the use of RdCVF and RdCVF2 for the treatment of neurological disease.

## Authors' contributions

EC conceived, carried out experiments, analyzed the data and wrote the manuscript. NW analyzed the data and designed the regionalization. SMS analyzed the data and advised on stereology. OP conceived the regionalization tool. J-AS conceived the experiments. TL conceived, carried analyzed the data and wrote the manuscript. All authors read and approved the manuscript.

## Author's information

EC, Research Engineer Inserm, NW Assistant Professor U. Strasbourg, SMS, Associate Professor UPMC, OP Research Director CNRS, JAS, Professor UPMC and TL Research Director Inserm.

## Pre-publication history

The pre-publication history for this paper can be accessed here:

http://www.biomedcentral.com/1471-2415/11/38/prepub

## Supplementary Material

Additional file 1 **℮-CONOME journals**. **(a) **ACQUISITION.JNL. **(b) **COUNTING.JNL. Bold words correspond to recorded journals. All the numbered lanes designate building functions use by the journals. Italic words indicate assigned variables of the journals. Green words in italic are not used in the ℮-CONOME application. Click here for file

Additional file 2 **Illustration of the image processing: (a) before processing. (b) after processing**. The yellow squares indicate the cones. The parameters for counting are indicated in green. Click here for file

Additional file 3 **Counting of cones**. **(a) **Stereological method: serial 1. **(b) **Stereological method: serial 2. **(c) **Global automated method: serial 1. **(d) **Global automated method: serial 2. **(e) **Automated stereological method. Click here for file

Additional file 4 **Comparison of the three methods of counting before normalization (BN) and after (AN)**. **(a) **Stereological method. **(b) **Global automated method. (**C**) Automated stereological method. Click here for file

Additional file 5 **Sections of the C57BL/6@N retina at PN 35 after labeling with S-opsin antibodies (green)**. PNA, red, DAPI, blue. **(a) **dorsal. **(b) **ventral. Notice that labeling was made with a different aliquot of the S-opsin antibodies as compared to figure 6. Scale bar 25 μm. Click here for file
