# Supplementary material for: ℮-conome: an automated tissue counting platform of cone photoreceptors for rodent models of retinitis pigmentosa
Source: BMC Ophthalmol. 2011 Dec 20;11:38. doi: 10.1186/1471-2415-11-38 (PMC3271040; doi:10.1186/1471-2415-11-38)
Supplement: Additional file 1 — ℮-CONOME journals. (a) ACQUISITION.JNL. (b) COUNTING.JNL. Bold words correspond to recorded journals. All the numbered lanes designate building functions use by the journals. Italic words indicate assigned variables of the journals. Green words in italic are not used in the ℮-CONOME application. [file 1471-2415-11-38-S1.DOC]

**Additional file 1a: ACQUISITION.JNL**

**I: SAVEIMAGE**

**Run Journal["MOVEZ SUR CEL"]**

**1.** Device.Focus.CurPos = "Z"+str[ret]

**2.** Delay[2,SECONDS]

**Run Journal C:\retine\journals\ACQ EXPLAN\MOVEZx40.JNL**

**3.** Delay[10,MILLISEC]

**Run Journal["autofocus2"] See details on page 2 (II: AUTOFOCUS-2)**

*Device.Focus.Top = FOCUS+4*

*Device.Focus.Bottom = FOCUS-4*

*Device.Focus.Home = FOCUS*

*Trace [FOCUS]*

**4.** Legacy:MetaDevice Shutter - Open[]

**5.** Overwrite "Z" = Acquire Z Series[BOTTOM, TOP, HOME, USEPLANECOUNT, 9, '''','''']

**6.** Shutter - Close[]

**7.** Set Image Zoom[[Last Result],50]

**8.** im = "explan"+str[ret]10: Select Image[" "]

**9.** surface = Image.ZDistance

**10.** Icol = floor[[Region.left+Region.Width]/Region.Width]

**11.** ligne = floor[[Region.Top + Region.Height]/ Region.Height]-ligne0+1

**12.** ligne = floor([Region.Top + Region.Height]/ Region.Height]

**13.** cpt_L = floor((lligne]/26+1

**14.** cpt_ii = lligne -floor[lligne/26)*26+1

**15.** cpt_L = if[test>1,cpt_L = cpt_L+1, cpt_L]

**16.** col = floor[[Region.left]Region.Width]/Region.Width]

**17.** ligne = CHR[64+cpt_L]+CHR[96+cpt_II]

**18.** col = if[LEN[str[col]]=1,"0"+str[col]]

**19.** imname = "scan_"+ligne+str[col]

**20.** Select Image[" "]

**21.** Define Image Property[[Last Result], "ligne", 0]

**22.** Define Image Property[[Last Result], "col", 0]

**23.** Define Image Property[[Last Result], "surface", 0]

**24.** Image.ZDistance = surface

**25.** Image.StageLabel = ligne+str[col]

**26.** Image.Wavelength = 0

**27.** Image.ZAbsolute = surface

**28.** Preferences[]

**Run Journal["IMSAVE"]**

**29.** Run User Program["retine.UserMethods", "IMSAVE", NOKEEPINMEM]

IF boucle=1 THEN

**30.** Select Image[" "]

*ex = Image.FileDrive+image.FileDir+"explan.jpg"*

**32.** Select Image[" "]

Image.FilePath = ex

**33.** Select Region((Last Result], "11111111"]

**34.** Save[[Last Result]]

ELSE

END IF

boucle = boucle+1

**35.** SelectIMAGE[3 3]

*rep = Image.FilePath*

**36.** Run User Program["retine.UserMethods","exc", NOKEEPINMEM]

**37.** Overwrite "Maximum" = Stack Arithmetic["Z"]

**38.** Set Image Zoom["Maximum" = Stack Arithmetic["Z"]

**39.** Auto Threshold for Dark Objects[]

**41.** Overwrite [Source] = Clip Image["Maximum", 0]

**42.** Image/Plane with Zoom([Last Result]]

**43.** Copy[(Last Result])

**44.** im = "explan"+str[ret]

**45.** Select Image[" "]

**46.** Paste[[Last Result])

**47.** Close["Maximum"]

**48.** Close["Zoomed Copy of Maximum"]

***End of Journal***

**II: AUTOFOCUS-2**

**1.** "Noshutter" = Acquire - Load Setting[EXPOSURE and BINNING and REGION and ILLUMINATION and DISPLAY and SETTING NAME and SAVING and CORRECTION and CORRIMAGES and ANNOTATION and SPECIAL and COLOR SCALING]

**2.** Legacy: MetaDevice Shutter - Open[]

**3.** Camera. Digital.Exposure = 20

**4.** Auto Expose[]

**5.** Close[[Last Result]]

**6.** Find Focus[32,6,NOBACKLASH]

**7.** Adjust Focus[8, 3.33333, NOBACKLASH]

**8.** Shutter - Close[]

**9.** "retine NOS" = Acquire - Load Setting[EXPOSURE and BINNING and REGION and ILLUMINATION and DISPLAY and SETTING NAME and SAVING and CORRECTION and CORRIMAGES and ANNOTATION and SPECIAL and COLOR SCALING]

**10.** Auto Expose[]

**11.** Shutter - Close[]

**12.** Delay[25, MILLISEC]

*FOCUS = Device.Focus.CurPos*

*testz = [[[FOCUS<[z0+50] ] and [FOCUS>[Z0-50]]]*

*WHILE testz=0 DO*

*FOCUS = Device.Focus.CurPos*

**13.** Select Image[[10: Auto Expose]]

*FOCUS = Image.ZAbsolute*

*testz = [[[FOCUS<[z0+50] ] and [FOCUS>[Z0-50]]]*

*IF testz=0 THEN*

*FOCUS + Z0*

*testz= 1*

*ELSE*

*END IF*

*WEND*

**14.** Close [[10:Auto Expose]]

**15.** Legacy: MetaDevice Shutter - Open[]

***End of Journal***

**Additional file 1b: COUNTING.JNL**

**I: FINDSPOT\3D Measurements**

*Max0=0*

*Stereo=0*

*name =right[Image.Name.4]*

**Run Journal["Variables"]**

**1.** nompile = Image.FileName

**2.** Select Image[(Current At Start)]

*Z = Image.ZAbsolute*

*SurfMin = 100*

*SurfMax = 1000*

**3.** Open Summary Log[OPENFILE and OVERWRITEMODE, "best focus"]

**4.** Pause Summary Logging []

**5.** New "Best Focus" = Stack Arithmetic[(Current At Start])

**6.** Overwrite [6: Stack Aritmetic] = Basic Filters[[6: Stack Arithmetic],3, 3, 1]

**Run Journal C:\retine\3DvolMM6\FindSpots\auto_dark.JNL**

**7.** Threshold Image[(6: Stack Arithmetic), 1000, 4095, Inclusive]

**8.** Integrated Morphometry - Reset Current[]

**9.** Integrated Morphometry - Reset Filters[]

**10.** Integrated Morphometry - Load State["best focus"]

**11.** Integrated Morphometry - Measure[[6: Stack Arithmetic],-1]

**12.** Show Region Statistics [[Last Result], ENTIREIMAGE]

**13.** Integrated Morphometry - Log Data[[6: Stack Arithmetic],SUMMARY,CURRENTDATA,1,2]

*Fd = ShowRegionStatistics.Minimum*

*max = IMASummary.Max*

*max = 0*

**14.** Integrated Morphometry - Reset Current []

**15.** Threshold Image["Best Focus" , 1000, 4095,Inclusive]

**16.** Adjust Digital Contrast[[6: Stack Arithmetic],ALL, 50, 50, 1.93

**17.** Show Region Statistics[[6: Stack Arithmetic], ENTIREIMAGE]

*BI = ShowRegionStatistics.Average*

**18.** Auto Threshold for Light Objects[Legacy heuristic algorithm]

**19**. Show Region Statistics[[6: Stack Arithmetic], ENTIREIMAGE]

*Cperc = ShowRegionStatistics.PctThresholdeArea*

**20.** Threshold Image["Best Focus", 1000, 4095,Inclusive]

**21.** Pause Summary Logging[]

**22.** Integrated Morphometry - Measure["Best Focus" ,-1]

**23.** Integrated Morphometry - Log Data["Best Focus", SUMMARY, CURRENTDATA,1,2]

**24.** Resume Summary Logging[]

*Dcel = IMASummary.Count*

*Trace[["B="]str[BI]+ " C="+str[Cperc]+" D="+str[Dcel]]]]*

**25.** Integrated Morphometry - Clear Object Overlay[[6: Stack Arithmetic)]

IF [BI< B]AND[Cperc<C] THEN

TEST_Filtre = 1

ELSE

IF Dcel>D THEN

TEST_Filtre = 1

ELSE

TEST_Filtre = 0

END IF

END IF

**26.** Close Summary Log[]

IF max=0 THEN

**27.** Set Image Zoom[[Last Result], 50]

**28.** Threshold Image[[Last Result],0,0]

**29.** Integrated Morphometry - Reset Current []

**30.** Set Image Zoom[[Last Result],100]

**31.** New "Overlay Images" = As Displatyed[[Last Result]]

*comptage = 0*

*count = 0*

count = 0

comptage = "Excl"

**32.** Close[[Current At Start]]

IF [Fd<FondMax] THEN

IF[MAX< AMAS]AND[DARK<DARK8MAX]AND[test8fILTRE+1] then

**33.** Threshold Image[[6: Stack Aritmetic], 1000,4095,Off]

**34.** Select Image[[Current At Start]]

IF rapide=0 THEN

**Run Journal C:\retine\3DvolMM6\FindSpots\spots3D.JNL**

**35.** Close[["Close-Open"]

**36.** Close[["Close-Open-2"]

**37.** Close["Maximum-2"]

**38.** Close["Maximum"]

**39.** Close["No Neighbors"]

**40.** Close[[Current At Start]]

ELSE

IF rapide=1 THEN

**41.** Overwrite"FAST" = Stack Arithmetic[[Current At Start]]

**Run Journal C:\retine\3DvolMM6\FindSpots\spots3D Rapide.JNL**

**42.** Close["Close-Open"]

**43.** Close["Close-Open2"]

**44.** Close["FAST"]

**45.** Close["FAST"]

**46.** Close[[Current At Start]]

ELSE

IF rapide=2 THEN

**47.** Overwrite "FAST" = Stack Arithmetic[[Current At Start]]

**Run Journal C:\retine\3DvolMM6\FindSpots\spots3D Rapide2.JNL**

**48.** Close["Close-Open"]

**49.** Close["Close-Open-2"]

**50.** Close["NN"]

**51.** Close["FAST"]

**52.** Close[[Current At Start]]

ELSE

END IF

END IF

END IF

ELSE

**53.** Set Image Zoom[[Last Result],100]

**54.** Threshold Image[(Last Result],0,0]

**55.** Integrated Morphometry - Reset Current[]

**56.** New "Overlay images" = As Displayed[[6: Stack Arithmetic]]

*count = 0*

*comptage = "Excl"*

**57.** Text[[Last Result],4, 19,0,0,255,MS Sans Serif, Regular,24,"%comptage% [%count%] %name%dark_max=%dark%"]

**58.** Close[[6: Stack arithmetic]]

**59.** Close[[Current At Start]]

END IF

ELSE

**60.** Set Image Zoom[[Last Result],50]

**61.** Threshold Image[[Last Result], 0,0]

**62.** Integrated Morphometry - Reset Current[]

**63.** Set Image Zoom[[Last Result], 100]

**64.** New "Overlay Images" = As Displayed[[Last Result]]

**65.** *comptage = 0*

**66.** *count = 0*

count = 0

comptage = "Excl"

**67.** Close'(Current At Start]]

END IF

**68.** Define Image Property'[Last Result], "comptage", "%comptage%"]

**69.** Define Image Property[[Last Result], "col", "%col%"]

**70.** Define Image Property[[Last Result], "ligne", "%ligne%"]

**71.** Define Image Property[[Last Result], "Z", "%Z%"]

**72.** Image.Annotation = str[comptage]

**73.** Image.Wavelength = comptage

**74.** Image.IllumSetting = comptage

**75.** Text'(Last Result],4400,0,0,255,MS Sans Serif,Regular,24"Fond=%Fd%

**76.** Adjust Digital Contrast["Overlay Images",ALL,50,50,1,19]

IF ImageExists["Best Focus"] THEN

**77.** Close["Best Focus"]

ELSE

END IF

***End of Journal***

**II: Editor\variables** (* Value of the variables used in the study)

1. B = Best Focus average intensity

B = xxxx (=4000)*

1. C = Pourcentage Best Focus Threshold autothreshold area percentage

C = xxx (=100)*

1. D = Nombre de cellules détectées sur Best Focus avant traitement

D = n+1

1. Spotcutoff = xx (=30)*
2. Spotsize = xx (=19)*
3. SurfSpot = xx (=30)*
4. IntMin = x (=0)*
5. NBObjectsMin = n
6. FondMax = xxxx (=1250)*
7. Cluster = 10^x^ (=10^8^)*
8. Dark_max = 10^x^ (=10^3^)*
9. Rapide = 0 Mesure 3D Normal
10. Rapide = 1 Mesure 3D rapide
11. rapide = 1

***End of Journal***
