# Supplementary figures and images for: ℮-conome: an automated tissue counting platform of cone photoreceptors for rodent models of retinitis pigmentosa
Source: BMC Ophthalmol. 2011 Dec 20;11:38. doi: 10.1186/1471-2415-11-38 (PMC3271040; doi:10.1186/1471-2415-11-38)

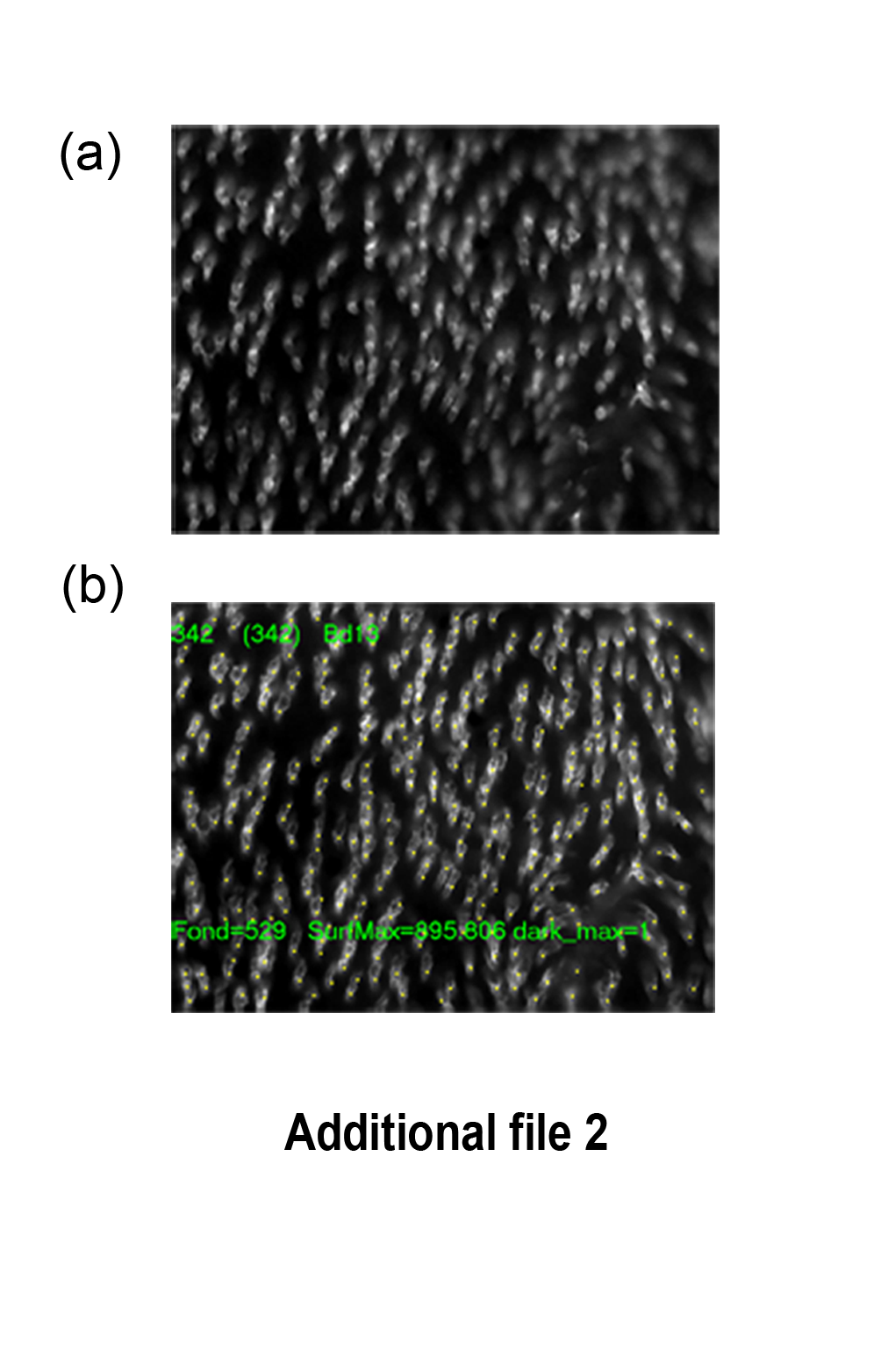

Supplement: Additional file 2 — Illustration of the image processing: (a) before processing. (b) after processing. The yellow squares indicate the cones. The parameters for counting are indicated in green. [file 1471-2415-11-38-S2.TIFF]

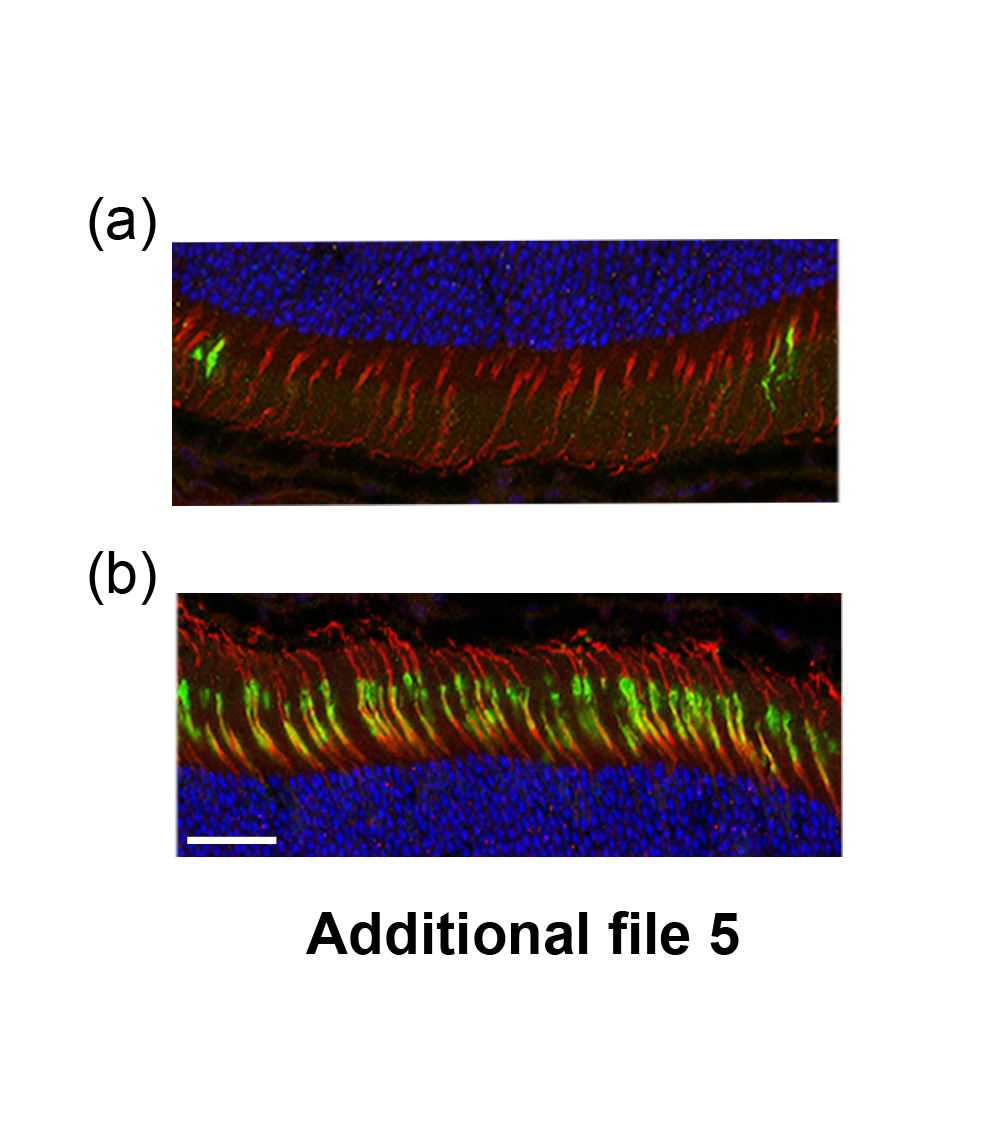

Supplement: Additional file 5 — Sections of the C57BL/6@N retina at PN 35 after labeling with S-opsin antibodies (green). PNA, red, DAPI, blue. (a) dorsal. (b) ventral. Notice that labeling was made with a different aliquot of the S-opsin antibodies as compared to figure 6. Scale bar 25 μm. [file 1471-2415-11-38-S5.TIFF]
