# Supplementary material for: ℮-conome: an automated tissue counting platform of cone photoreceptors for rodent models of retinitis pigmentosa
Source: BMC Ophthalmol. 2011 Dec 20;11:38. doi: 10.1186/1471-2415-11-38 (PMC3271040; doi:10.1186/1471-2415-11-38)
Supplement: Additional file 3 — Counting of cones. (a) Stereological method: serial 1. (b) Stereological method: serial 2. (c) Global automated method: serial 1. (d) Global automated method: serial 2. (e) Automated stereological method. [file 1471-2415-11-38-S3.DOC]

| **a** | **15 days** | | **35 days** | | | **43 days** | | | **60 days** | | | **90 days** | | |
| --- | --- | --- | --- | --- | --- | --- | --- | --- | --- | --- | --- | --- | --- | --- |
|  | **wt** | ***rd1*** |  | **wt** | ***rd1*** |  | **wt** | ***rd1*** |  | **wt** | ***rd1*** |  | **wt** | ***rd1*** |
| **1** | 6602 | 7268 |  | 6671 | 4188 |  | 8149 | 3583 |  | 7963 | 2848 |  | 7759 | 2280 |
| **2** | 7820 | 6057 |  | 7400 | 4745 |  | 7485 | 2997 |  | 7623 | 2712 |  | 6757 | 2375 |
| **3** | 7211 | 5751 |  | 6707 | 4439 |  | 6984 | 4248 |  | 7224 | 2899 |  | 6703 | 1241 |
| **4** | 8564 | 6815 |  | 7990 | 5042 |  | 7707 | 3968 |  | 7966 | 2315 |  | 6769 | 1076 |
| **5** | 8350 | 7489 |  | 6913 | 5383 |  | 7020 | 3334 |  | 7533 | 2637 |  | 7561 | 1213 |
| **6** | 9567 | 5569 |  | 9396 | 4665 |  | 6939 | 3673 |  | 6939 | 2965 |  | 7434 | 1355 |
| **7** | 8941 | 10665 |  | 7755 | 5665 |  | 7780 | 2817 |  | 7039 | 2097 |  | 10370 |  |
| Mean | 8151 | 7088 |  | 7547 | 4875 |  | 7438 | 3517 |  | 7470 | 2639 |  | 7622 | 1590 |
| SEM | 385 | 659 |  | 364 | 197 |  | 178 | 193 |  | 158 | 122 |  | 486 | 236 |
| Decrease  in cones  % |  |  |  |  | 31.2 |  |  | 50.4 |  |  | 62.8 |  |  | 77.6 |
| **b** | **15 days** | | **35 days** | | | **43 days** | | | **60 days** | | | **90 days** | | |
|  | **wt** | ***rd1*** |  | **wt** | ***rd1*** |  | **wt** | ***rd1*** |  | **wt** | ***rd1*** |  | **wt** | ***rd1*** |
| **1** | 7396 | 6564 |  | 7677 | 5557 |  | 7863 | 4003 |  | 8227 | 2514 |  | 7520 | 1088 |
| **2** | 9083 | 8372 |  | 6718 | 4824 |  | 7207 | 3745 |  | 7629 | 3151 |  | 7601 | 1315 |
| **3** | 8020 | 6724 |  | 6780 | 3991 |  | 6992 | 3870 |  | 6745 | 2948 |  | 7262 | 1065 |
| **4** | 9647 | 8490 |  | 9689 | 4719 |  | 7147 | 3265 |  | 8057 | 2870 |  | 8113 | 1183 |
| **5** | 8209 | 7693 |  | 7988 | 5474 |  | 7224 | 3887 |  | 6467 | 2894 |  | 6777 | 1002 |
| **6** | 7180 | 9239 |  | 6639 | 4368 |  | 5714 | 3929 |  | 5672 | 3061 |  | 6541 | 1224 |
| **7** | 7510 | 6829 |  | 7449 | 4665 |  | 6519 | 3358 |  | 6723 | 2394 |  | 6939 | 1295 |
| **8** |  |  |  |  | 4058 |  |  |  |  |  |  |  |  | 1463 |
| Mean | 8149 | 7702 |  | 7563 | 4707 |  | 6952 | 3722 |  | 7074 | 2833 |  | 7251 | 1204 |
| SEM | 347 | 392 |  | 405 | 206 |  | 255 | 110 |  | 351 | 105 |  | 205 | 54 |
| Decrease  in cones  % |  |  |  |  | 38.9 |  |  | 51.7 |  |  | 63.2 |  |  | 84.4 |

| **c** | **15 days** | | **35 days** | | | **43 days** | | | **60 days** | | | **90 days** | | |
| --- | --- | --- | --- | --- | --- | --- | --- | --- | --- | --- | --- | --- | --- | --- |
|  | **wt** | ***rd1*** |  | **wt** | ***rd1*** |  | **wt** | ***rd1*** |  | **wt** | ***rd1*** |  | **wt** | ***rd1*** |
| **1** | 4595 | 8352 |  | 6604 | 4062 |  | 7460 | 3185 |  | 6056 | 2794 |  | 7331 | 948 |
| **2** | 4988 | 4983 |  | 6396 | 3480 |  | 6825 | 3409 |  | 6893 | 2771 |  | 5347 | 1266 |
| **3** | 6365 | 8009 |  | 7929 | 4095 |  | 6278 | 3003 |  | 6735 | 3179 |  | 5045 | 1363 |
| **4** | 6265 | 7597 |  | 8068 | 4035 |  | 7379 | 3791 |  | 7001 | 2658 |  | 6287 | 1287 |
| **5** | 6425 | 7790 |  | 7487 | 4384 |  | 6917 | 2866 |  | 7341 | 2499 |  | 6056 | 948 |
| **6** | 7340 | 7969 |  | 7060 | 3708 |  | 7676 | 2841 |  | 6679 | 3730 |  | 6964 |  |
| **7** | 7970 | 6246 |  | 6371 | 3217 |  | 7066 | 3267 |  | 7317 | 2271 |  | 6246 |  |
| **8** |  |  |  |  |  |  | 7040 |  |  |  |  |  |  |  |
| Mean | 6278 | 7278 |  | 7131 | 3854 |  | 7080 | 3195 |  | 6860 | 2843 |  | 6182 | 1162 |
| SEM | 450 | 459 |  | 269 | 153 |  | 154 | 127 |  | 166 | 182 |  | 307 | 89 |
| Decrease  in cones  % |  |  |  |  | 48.8 |  |  | 59.1 |  |  | 62.2 |  |  | 84.6 |
| **d** | **15 days** | | **35 days** | | | **43 days** | | | **60 days** | | | **90 days** | | |
|  | **wt** | ***rd1*** |  | **wt** | ***rd1*** |  | **wt** | ***rd1*** |  | **wt** | ***rd1*** |  | **wt** | ***rd1*** |
| **1** | 5722 | 6906 |  | 8795 | 3652 |  | 7110 | 3455 |  | 6726 | 3526 |  | 7091 | 1880 |
| **2** | 4736 | 6631 |  | 6748 | 3842 |  | 7557 | 3264 |  | 7657 | 3354 |  | 7357 | 1729 |
| **3** | 5335 | 7791 |  | 7233 | 3720 |  | 7052 | 3772 |  | 7031 | 2864 |  | 7354 | 1545 |
| **4** | 6949 | 7430 |  | 6926 | 3726 |  | 6297 | 3295 |  | 6491 | 2839 |  | 6457 | 1584 |
| **5** | 6010 | 7655 |  | 7134 | 3533 |  | 7696 | 3211 |  | 5844 | 2842 |  | 7393 | 1254 |
| **6** | 4953 | 6835 |  | 7245 | 3645 |  | 6983 | 3791 |  | 5941 | 3452 |  | 6962 | 1656 |
| **7** | 6023 | 5698 |  | 7615 |  |  | 7817 | 4143 |  | 6133 | 3177 |  |  | 1451 |
| **8** |  | 6559 |  |  |  |  |  |  |  |  |  |  |  | 1521 |
| Mean | 5675 | 6938 |  | 7385 | 3686 |  | 7216 | 3562 |  | 6546 | 3151 |  | 7102 | 1578 |
| SEM | 284 | 242 |  | 256 | 42 |  | 198 | 132 |  | 246 | 114 |  | 147 | 66 |
| Decrease  in cones  % |  |  |  |  | 50.1 |  |  | 50.5 |  |  | 56.3 |  |  | 78.1 |

| **e** | **15 days** | | **35 days** | | | **43 days** | | | **60 days** | | | **90 days** | | |
| --- | --- | --- | --- | --- | --- | --- | --- | --- | --- | --- | --- | --- | --- | --- |
|  | **wt** | ***rd1*** |  | **wt** | ***rd1*** |  | **wt** | ***rd1*** |  | **wt** | ***rd1*** |  | **wt** | ***rd1*** |
| **1**  **2**  **3**  **4**  **5**  **6**  **7**  **8** | 4902  4274  5291  4682  5266  6446 | 6655  4114  6424  6659  6097  6241  6446 |  | 5536  5509  6575  6593  6278  5910  5272 | 3360  2683  3274  3336  3616  2436  2991  2615 |  | 5551  6065  5667  6396  5828  4888  6156 | 1800  1795  1748  2192  2073  1899  2204 |  | 5053  5663  5492  5924  5503  6054  6104 | 1304  1536  1813  1628  1694  1679 |  | 6037  4488  4431  5313  4962  5980  5297 | 1074  968  781  1014  1056 |
| Mean | 5144 | 6092 |  | 5953 | 3039 |  | 5793 | 1959 |  | 5685 | 1609 |  | 5215 | 979 |
| SEM | 303 | 339 |  | 203 | 150 |  | 187 | 74 |  | 141 | 71 |  | 243 | 53 |
| Decrease )  in cones  % |  |  |  |  | 50.1 |  |  | 67.8 |  |  | 73.6 |  |  | 83.9 |

**Additional file 3: Counting of cones**

a: Stereological method: serial 1

b: Stereological method: serial 2

c: Global automated method: serial 1

d: Global automated method: serial 2

e: Automated stereological Method
