# Supplementary material for: ℮-conome: an automated tissue counting platform of cone photoreceptors for rodent models of retinitis pigmentosa
Source: BMC Ophthalmol. 2011 Dec 20;11:38. doi: 10.1186/1471-2415-11-38 (PMC3271040; doi:10.1186/1471-2415-11-38)
Supplement: Additional file 4 — Comparison of the three methods of counting before normalization (BN) and after (AN). (a) Stereological method. (b) Global automated method. (C) Automated stereological method. [file 1471-2415-11-38-S4.DOC]

| **a** | **15 days** | | **35 days** | | | **43 days** | | | **60 days** | | | **90 days** | | |
| --- | --- | --- | --- | --- | --- | --- | --- | --- | --- | --- | --- | --- | --- | --- |
|  | **BN** | **AN** |  | **BN** | **AN** |  | **BN** | **AN** |  | **BN** | **AN** |  | **BN** | **AN** |
|  | 7268 | 7228 |  | 4188 | 3267 |  | 3583 | 2889 |  | 2848 | 2479 |  | 2375 | 2137 |
|  | 6057 | 6632 |  | 4745 | 3924 |  | 2997 | 2600 |  | 2712 | 2387 |  | 1241 | 1174 |
|  | 5751 | 6031 |  | 4439 | 3587 |  | 4248 | 3388 |  | 2899 | 2520 |  | 1076 | 935 |
|  | 6815 | 7010 |  | 5042 | 4126 |  | 3968 | 3142 |  | 2315 | 1963 |  | 1213 | 1043 |
|  | 7489 | 7384 |  | 5383 | 4271 |  | 3334 | 2775 |  | 2637 | 2308 |  | 1355 | 1212 |
|  | 5569 | 4438 |  | 4665 | 3713 |  | 3673 | 2988 |  | 2097 | 1511 |  |  |  |
|  | 10665 | 8559 |  | 5665 | 4921 |  | 2817 | 2435 |  |  |  |  |  |  |
| Mean | 7088 | 6754 |  | 4875 | 3973 |  | 3517 | 2888 |  | 2585 | 2195 |  | 1452 | 1304 |
|  |  |  |  |  |  |  |  |  |  |  |  |  |  |  |
|  |  |  |  |  |  |  |  |  |  |  |  |  |  |  |
| **b** | **15 days** | | **35 days** | | | **43 days** | | | **60 days** | | | **90 days** | | |
|  | **BN** | **AN** |  | **BN** | **AN** |  | **BN** | **AN** |  | **BN** | **AN** |  | **BN** | **AN** |
|  | 8352 | 8559 |  | 4062 | 4126 |  | 3185 | 2889 |  | 2794 | 2435 |  | 948 | 1511 |
|  | 4983 | 4921 |  | 3480 | 3388 |  | 3409 | 3267 |  | 2771 | 2387 |  | 1266 | 1174 |
|  | 8009 | 7384 |  | 4095 | 4271 |  | 3003 | 2600 |  | 3179 | 2775 |  | 1363 | 1512 |
|  | 7597 | 6632 |  | 4035 | 3924 |  | 3791 | 3713 |  | 2658 | 2308 |  | 1287 | 1233 |
|  | 7790 | 7010 |  | 4384 | 4438 |  | 2866 | 2520 |  | 2499 | 2137 |  | 948 | 981 |
|  | 7969 | 7228 |  | 3708 | 3587 |  | 2841 | 2479 |  | 2271 | 1963 |  |  |  |
|  | 6246 | 6031 |  | 3217 | 2988 |  | 3267 | 3142 |  |  |  |  |  |  |
| Mean | 7278 | 6823 |  | 3854 | 3817 |  | 3195 | 2944 |  | 2695 | 2334 |  | 1162 | 1179 |
|  |  |  |  |  |  |  |  |  |  |  |  |  |  |  |
|  |  |  |  |  |  |  |  |  |  |  |  |  |  |  |
|  |  |  |  |  |  |  |  |  |  |  |  |  |  |  |
| **c** | **15 days** | | **35 days** | | | **43 days** | | | **60 days** | | | **90 days** | | |
|  | **BN** | **AN** |  | **BN** | **AN** |  | **BN** | **AN** |  | **BN** | **AN** |  | **BN** | **AN** |
|  | 6665 | 7384 |  | 3360 | 4438 |  | 1800 | 2600 |  | 1304 | 1963 |  | 1074 | 1511 |
|  | 4114 | 4921 |  | 2683 | 3713 |  | 1795 | 2520 |  | 1536 | 2137 |  | 968 | 1043 |
|  | 6424 | 7010 |  | 3274 | 4126 |  | 1748 | 2479 |  | 1813 | 2775 |  | 781 | 935 |
|  | 6659 | 8559 |  | 3336 | 4271 |  | 2192 | 3142 |  | 1628 | 2308 |  | 1014 | 1174 |
|  | 6097 | 6031 |  | 2436 | 3388 |  | 2073 | 2988 |  | 1694 | 2435 |  | 1056 | 1233 |
|  | 6241 | 6632 |  | 2991 | 3924 |  | 1899 | 2889 |  | 1679 | 2387 |  |  |  |
|  | 6446 | 7228 |  | 2615 | 3524 |  | 2204 | 3267 |  |  |  |  |  |  |
|  |  |  |  |  |  |  |  |  |  |  |  |  |  |  |
| Mean | 6092 | 6823 |  | 2956 | 3911 |  | 1959 | 2841 |  | 1609 | 2334 |  | 979 | 1179 |

**Additional file 4**: Comparison of the three methods of counting before (BN) and after normalization (AN)

a: Stereological method

b: Global automated method

c: Automated stereological Method
